# Commit to your putting stroke: exploring the impact of quiet eye duration and neural activity on golf putting performance

**DOI:** 10.3389/fpsyg.2024.1424242

**Published:** 2024-07-11

**Authors:** Laura M. Carey, Georgia Alexandrou, Simon Ladouce, Dimitrios Kourtis, Marika Berchicci, Angus M. Hunter, David I. Donaldson

**Affiliations:** ^1^Health and Life Sciences, University of the West of Scotland, Glasgow, United Kingdom; ^2^Institute of Social Marketing and Health, University of Stirling, Stirling, United Kingdom; ^3^Brain & Cognition, KU Leuven, Leuven, Belgium; ^4^Psychology and Natural Sciences, University of Stirling, Stirling, United Kingdom; ^5^Department of Psychological, Health and Territorial Sciences, G. d'Annunzio University of Chieti and Pescara, Chieti, Italy; ^6^Department of Sport Science, Nottingham Trent University, Nottingham, United Kingdom; ^7^School of Psychology and Neuroscience, University of St Andrews, St Andrews, United Kingdom

**Keywords:** golf putting, expertise, quiet eye, EEG, performance

## Abstract

**Introduction:**

There is a growing interest in characterizing the cognitive-motor processes that underlie superior performance in highly skilled athletes. The aim of this study was to explore neural markers of putting performance in highly skilled golfers by recording mobile EEG (electroencephalogram) during the pre-shot period.

**Methods:**

Twenty-eight right-handed participants (20 males) with a mean age of 24.2 years (± 6.4) and an average handicap of +1.7 (± 6.4) completed a testing session. Following the warm-up, participants completed 140 putts from a distance of 8ft (2.4m), with putts taken from 5 different positions. While putting, participants wore an eye tracker and a gel-based EEG system with 32 electrodes. Time and frequency domain features of the EEG signals were extracted to characterize Movement-Related Cortical Potentials (MRCP) and rhythmic modulations of neural activity in theta, alpha, sensorimotor and beta frequency bands associated with putting performance.

**Results:**

Eye-tracking data demonstrate that mean Quiet Eye durations are not a reliable marker of expertise as the same duration was found for both successful and unsuccessful putts. Following rigorous data processing data from 12 participants (8 males, mean age 21.6 years ± 5.4, average handicap +1.5 ± 4.4) were included in the EEG analysis. MRCP analysis revealed performance-based differences, with unsuccessful putts having a greater negative amplitude in comparison to successful putts. Time frequency analysis of the EEG data revealed that successful putts exhibit distinct neural activity profiles compared to unsuccessful ones. For successful putts, greater suppression of beta was present in the central region prior to the putt. By contrast, increased frontal theta power was present for unsuccessful putts immediately before the putt (consistent with hesitation and the need for motor plan adjustments prior to execution).

**Discussion:**

We propose that neural activity may provide plausible insights into the mechanisms behind why identical QE durations can lead to both success and failure. From an applied perspective, this study highlights the merits of a multi-measure approach to gain further insights into performance differences within highly skilled golfers. We discuss considerations for future research and solutions to address the challenges related to the complexities of collecting clean EEG signals within naturalistic sporting contexts.

## 1 Introduction

Putting constitutes a fundamental aspect of the sport of golf, wherein a putter is required to strike the ball into the hole when it lies on (or just short of) the green. From a practical standpoint, proficient putting is paramount, due to its significant impact on overall performance and subsequent success (Baugher et al., 2016). From a scientific perspective, the nature of golf putting offers an ideal platform for investigating the cognitive processes underlying skilled performance. The process of putting involves a routine that makes it amenable to study; preceding the initiation of the putting action and the commencement of the backswing, there exists a phase of motor preparation during which the golfer assumes a static posture with the putter head positioned just behind the ball (referred to as the “address” in golf terminology). Investigating the processing that occurs during this pre-shot period, leading up to the putt, should furnish insights into the underlying cognitive and neural mechanisms governing action preparation (Gallicchio et al., 2017).

Over recent years, researchers investigating the putting motor preparation period have predominantly focused on investigating eye movements stillness, or Quiet Eye (QE), a metric derived from eye-tracking recordings. QE is defined as the final fixation or tracking gaze on a specific location that has an onset prior to the start of a final, critical movement (Vickers, 2007). When applied to golf putting, research has recommended maintaining a steady vision on the back of the ball (Vickers, 1996). Optimal QE duration is thought to involve the player keeping their eyes fixated on the ball for 2000–3000 ms before starting the backswing and throughout the stroke. After making contact, the player sustains this focus on the spot where the ball was for an additional 200 milliseconds, known as QE dwell time. Crucially, researchers have claimed that QE duration can differentiate between successful and less successful performances, even among experts (Wilson et al., 2016). However, these results are not unequivocal as Mann et al.’s (2011) found QE durations between successful and unsuccessful putts did not vary for both low and high handicap groups. Additionally, van Lier et al. (2010) discovered that optimal QE duration (defined to have ended when initiating the backswing) was not associated with performance. Similarly, when practitioners have tried to apply these findings, with elite golfers, results have been mixed (Farrow and Panchuk, 2016). In particular, it has proved difficult to explain why, across multiple putts, the same QE duration can lead to both success and failure (Farrow and Panchuk, 2016). Consequently, in an effort to gain greater insight into the processes supporting successful putting, the current study investigates performance using a multi-methods approach that combines eye tracking with a measure of neural activity derived from scalp recorded EEG.

Investigating neural activity within the pre-motor preparation phase has already shown some promise as a method for discriminating between successful and unsuccessful performance. Currently, the brain waves mainly explored in golf putting in the frequency domain are the theta band (4–7 Hz), the alpha band (8–12 Hz), the beta band (12–30 Hz), and the sensorimotor rhythm (SMR: 12–15 Hz). Superior golf putting performance has been linked to changes in relative theta power (Reinecke et al., 2011; Kao et al., 2013; Chen et al., 2022). For instance, Kao et al. (2013, 2014) discovered that midline theta power (i.e., FZ, CZ, PZ, OZ sites) was significantly lower for the best 15 putts compared to the worst 15 putts in a sample of professional and amateur golfers (handicap not stated, *n* = 12). Reinecke et al. (2011) observed that superior performance was associated with an increase in theta power over the left frontal scalp (electrode F3, however, only used F3, Fz, F4 in their analysis) with golfers who had an average of 7.9 土 6.4 handicap (considered immediate skilled, *n* = 20). Critically, as well as the differences in skill level, the definition of superior performance may have differed across the studies: the Kao et al. studies used holed putts, whereas, Reinecke et al. (2011) did not state a direct performance measure. Also, the timings of the epoch varied across these studies: Reinecke et al. (2011) used an average across the putting period (2 min), whereas Kao et al. used −3 s prior to initiation of the movement.

There are also mixed findings in studies employing neurofeedback training to encourage superior performance, revealing both a decrease in frontal midline theta (Fmθ) power in three highly skilled (handicap = 0) golfers (Kao et al., 2014) and a significant reduction in theta power (Chen et al., 2022). In contrast, superior performance without neurofeedback training was associated with a notable increase in theta power (Chen et al., 2022). Although Chen et al. (2022) did try and match the skill level across the group, the variation in skill level (reflected in the high standard error) within each group must be considered when interpreting the findings. For example, the function specific group (*n* = 12, mean handicap = 12.00 ± 11.02) exhibited much greater variation than either the traditional instruction group (*n* = 12, mean handicap = 14.00 ± 7.38) or the sham control group (*n* = 12, mean handicap = 18.00 ± 8.86). Nonetheless, taken together, the existing findings provide evidence that successful putting performance is associated with changes in theta power, specifically over frontal recording electrodes.

Following previous findings, the current study aims to gain clarity on the direction of the theta effect, and specific timings of the modulations throughout the pre-preparation period related to performance, when considering a sample of highly skilled golfers. Furthermore, through using the multi-measure approach we would like to gain insight into underlying cognitive and neural mechanisms governing action preparation (Gallicchio et al., 2017). For example, in golf putting, lower Fmθ levels may suggest reduced mental engagement, according to Kao et al. (2013, 2014) in professional and highly skilled golfers. A reduction in mental engagement seems in contrast to the response programming explanation (Williams et al., 2002) which is the dominant proposal as to how and why QE duration works (Walters-Symons et al., 2017). Aligned with the response program explanation, a longer QE enhances performance due to a longer period for cognitive programming (Vickers, 1996; Williams et al., 2002; Vickers, 2007). To help gain insight into the timings and potential link to QE durations, our study aims to explore fluctuations in theta power throughout the pre-putt preparation period using both the whole length of the pre-putt preparation period (−3 s) and at 500 ms time intervals.

Modulations in the alpha band have also been found to be associated with improved golf putting performance in a mixed sample of expert and novice golfers (Cooke et al., 2014). As with theta, however, there remains uncertainty regarding the direction of the alpha effect. For example, studies have reported both an increase (Baumeister et al., 2008) and a decrease (Babiloni et al., 2008; Cooke et al., 2014) in alpha power over frontocentral recording sites for successful compared to unsuccessful putts. It must be acknowledged that differences in skill level may be contributing to the ambiguity in the findings as the expert group in Baumeister et al. (2008) had large variations in skill level (average handicap = 8.3 ± 7.5). It could be argued the sample was more homogeneous in the Babiloni et al. (2008) and Cooke et al. (2014) studies, as participants in Babiloni et al. (2008) regularly competed in national and international competitions and practiced at least five times a week (no formal handicap was stated) and in Cooke et al. (2014), participants had a golf handicap < 5 (average handicap = 1.50 土 2.32). Discrepancies in findings may arise from variations in task design (e.g., examination of expert vs. novice/expert golfers), the specifics of the analysis (including epoch duration and electrode selection), and the specific analytical methods employed. It is important to note that in Cooke et al. (2014, 2015), the size of the hole was adjusted, and was reduced to half its original size for expert participants, whereas a standard hole size was used in Babiloni et al. (2008) and Baumeister et al. (2008). Another significant observation is that alpha modulation may change throughout the pre-shot period. For instance, Cooke et al. (2014) identified a two-phase pattern of alpha oscillations among expert golfers, characterized by an initial increase followed by a sudden decrease in alpha power in the last second before movement initiation. Our study, therefore, aims to explore fluctuations in alpha power throughout the pre-putt preparation period, examining the whole length of the pre-putt preparation period (−3 s) in 500 ms time intervals.

Successful performance has also been associated with a greater reduction in beta power in the last seconds preceding golf putts (Cooke et al., 2014). While these findings are from a single study (and one that only analyzed limited electrode sites F3, Fz, F4, C3, Cz, C4) they align with broader evidence suggesting a decrease in beta power relative to baseline in sensorimotor tasks, particularly in tasks requiring accuracy (Kilavik et al., 2013). It has been suggested that this reduction in beta power may reflect the activation of sensorimotor networks (Pfurtscheller and da Silva, 1999), indicating beta involvement in the planning, processing, and execution of actions, including their sensory and cognitive aspects (Pfurtscheller et al., 2003). Consequently, and following the findings of Cooke et al., in the present study we will examine changes in beta power throughout the pre-putt preparation period, but with a larger array of electrodes (31 channels) across the scalp.

To the best of the authors’ knowledge, the only studies examining sensorimotor rhythm (SMR) have been neurofeedback studies, including those by Cheng et al. (2015), who recruited 16 elite golfers (average handicap = 0 土 3.90), and Wu et al. (2024), who recruited 44 professional golfers. In both studies, SMR neurofeedback training was found to enhance performance, with participants who received the training exhibiting greater SMR power (at Cz for Cheng et al., 2015, and Cz & CPz for Wu et al., 2024) compared to the control group. Here it is notable that the samples examined are homogeneous across the two studies, which aids comparison and may have contributed to the consistency in findings. These results are encouraging, especially given there are differences in the methodologies employed between the two studies. Nonetheless, in Cheng et al. (2015) it remains uncertain whether putt distances might have influenced the outcomes, as they were individualized and not reported. This lack of standardization means that distances could have differed between the control and intervention groups. Additionally, performance in Cheng et al. (2015) was measured using error distance, rather than counting holed putts. In contrast, Wu et al. (2024) standardized the distance across all trials. Furthermore, they (Wu et al., 2024) assessed performance by asking participants to putt towards a hole and record the percentage of successful putts, which is more representative of competitive golfing scenarios. At this stage, further study is required to gain greater insight into SMR and performance.

Another form of electroencephalography (EEG) analysis that sheds light on the processes involved in planning and preparing voluntary motor movement is the Movement-Related Cortical Potentials (MRCP) (Shibasaki and Hallett, 2006). The change in amplitude of MRCPs over time is typically regarded as an index of motor preparation (Wright et al., 2012). The readiness potential (RP) is a marker of particular interest to study. The RP is an event-related potential that consists of a negative deflection in EEG that begins around 2 s before self-initiated movements (Shibasaki and Hallett, 2006). Two studies (Mann et al., 2011; Xu et al., 2021) have analyzed neural activity in golf putting using MRCP and RP relative to performance. The results have been inconsistent across the two studies, however, critically there were skill level differences within the participants recruited. Mann et al. (2011) included both experts (*n* = 10, average handicap = 1.20 土 1.23) and near-experts (n = 10, average handicap = 11.30 土 0.82), whereas Xu et al. (2021) examined 21 novice golfers. Mann et al. (2011) did not find any significant differences in MRCP amplitudes between successful and unsuccessful putts (analyzing C3, Cz, C4, P3, and P4 separately). By contrast, Xu et al. (2021) did report performance-based differences, with greater increased negativity for successful in comparison to unsuccessful putts; however, clear RP (Cz) were not evident in their figures presented. In addition, both of these studies used electrooculogram (EOG) data to measure gaze behavior (rather than an eye tracker). There were, however, substantial differences in the putting paradigm employed across these studies. In Mann et al. (2011) the golfers putted to a standardized hole from 12 ft., whereas in Xu et al. (2021) golfers putted the ball into a modified hole from 2 m. In this case the center of the hole had a radius of 5 cm rather than the standard 10.4 cm. Outside the hole, however, there were three imposed concentric circles with radii of 10, 15, and 20 cm. A “hit” was recorded if the golf ball went into the hole or circle and a “miss” was recorded if the golf ball went outside the outermost circle to balance the ratio of the two conditions. At this stage, given the methodological inconsistencies and the variation of skill level further research with a homogenous sample of expert golfers is merited before conclusions can be drawn.

Our study aims to assess whether QE duration and neural activity can be used as reliable markers associated with successful putting in highly skilled golfers. This study therefore addresses two specific hypotheses: (i) there will be a difference in QE duration as a function of performance, and (ii) successful performances will be distinguishable from unsuccessful performance based on neural activity. Given our interest in highly skilled golfers, our theoretical starting point for the expertise-based differences in neural activity was informed by the neural efficiency framework (Del Percio et al., 2009) and previous research. We therefore predicted that successful performance would be associated with greater suppression of frontal theta, an increase in alpha power (high band 10–13 Hz), greater suppression of beta (Cooke et al., 2014) and an increase in SMR power. In addition, for the RP, we predicted that performance related differences would be observed, with less negativity for successful putts in comparison to unsuccessful putts.

## 2 Materials and methods

### 2.1 Participants

Twenty-eight participants (20 males, 8 females), all of whom were right-handed, with normal or corrected vision, were included in the study. The mean age of the participants was 24.2 years (±6.4), and the average handicap was +1.7 (±6.4). On average, participants had been playing golf for 12.8 years (±5.69), practiced for 15.5 h per week (±11.5), made 31.3 putts per round (±2.84), achieved greens in regulation 56.2% of the time (±10.1), and scored an average of 85% (±21.1) from 6 feet straight. For the sample of 12 participants included in the analysis of the EEG data (4 females, mean age 21.6 years ±5.4, average handicap +1.5 ± 4.4) participants had been playing golf for an average of 12.2 years (±6.54), practiced for 16 h per week (±12.5), made 31.1 putts per round (±3.10), achieved greens in regulation 57% of the time (±10.6), and scored an average of 88% (±21.6) from 6 feet straight.

### 2.2 Protocol

**Figure 1:**
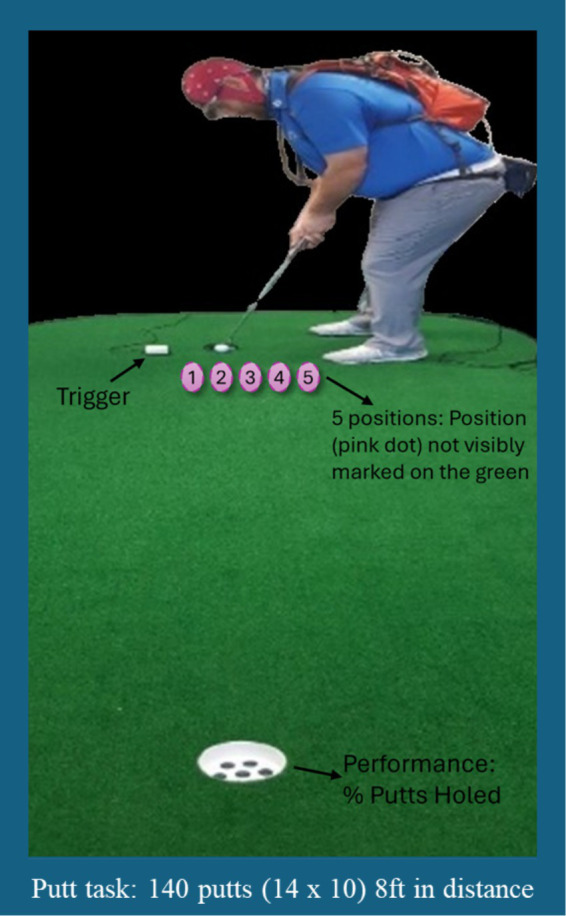
An image of a participant using the mobile equipment (eye tracking and EEG) whilst completing the putting task.

Participants attended testing sessions individually. They were fitted with a mobile eye tracker (ASL XG Mobile Eye Tracker) and EEG system comprising 32 Ag/AgCl electrodes fitted in an elastic cap according to the 10–20 International montage and connected to a portable amplifier (ANT-neuro, Enschede, The Netherlands). Calibration of the eye tracker was performed using five colored markers positioned near the participant’s feet while standing in a putting posture and addressing a golf ball. During calibration, participants were instructed to adopt a normal putting stance and maintain their gaze steady on the center of each marker, in a pre-designated order, for a duration of 100–200 ms. Participants used their own putter and Srixon AD333 Tour golf balls throughout the eye tracker calibration and the putting task. At the beginning of the putting task, participants completed a standardized warm-up protocol consisting of 12 practice putts, including 6 straight and 6 sloped putts, on an indoor artificial surface with a stimp meter rating of 10.2. Following the warm-up, participants completed a putting task (see Figure 1) involving 140 straight putts taken from a distance of 8 feet (2.4 m) from 5 different putt positions (5 cm apart). The putts were taken in blocks of 10 and randomization was applied within each of the seven blocks, with the constraint that they putted twice from each location in each block of ten putts. Each participant had a different order. The putt position was marked on the surface with a UV light so there were no obvious markings on the putt surface to slow down the learning of the positions. Re-calibration of the eye tracker occurred at the start of each putting block and whenever necessary (e.g., after a pupil recognition loss >100 ms or if the calibration had been lost).

### 2.3 Measures

#### 2.3.1 Task performance

Performance was assessed by the number of successful (holed) putts. Professional golfers, on average, have a probability rate of 50% success from 8 ft (PGA Tour, 2024).

#### 2.3.2 Quiet eye measures

Visual search behaviors were examined using EyeVision software (ASL Results Pro Analysis, formerly Argus, ASL) installed on a laptop (Dell Inspiron6400) captured at a frame rate of 30 Hz. All analyses were conducted post-testing. The onset of Quiet Eye (QE) had to occur before movement initiation of the backswing but could continue through the putting movement (Causer et al., 2017). QE offset was determined when gaze deviated from the target (ball or fixation marker) by more than 3° of visual angle for longer than 100 ms (Vickers, 2007).

#### 2.3.3 EEG features

EEG data were recorded with a sampling rate of 500 Hz, a 0.016–250 Hz bandpass filter, and a notch filter set at 50 Hz. The electrode AFz served as the ground and CPz as a common reference site. Electrode impedance was measured prior to each recording session and set below 10 kΩ using electrode gel. Similarly, impedances were checked throughout the session to maintain <10 kΩ. To timestamp the event of contact between the ball and putter, an acoustic box was connected to the EEG amplifier and a trigger code was sent via an acoustic box designed to capture the sound when the putter made contact with the ball. Although identifying the point of contact as time point zero means that some movement will be included within the pre-shot epoch, the alternative of timestamping the initial onset of movement is not sufficient for capturing the QE period (which onsets before movement initiation and continues after movement initiation (cf. Walters-Symons et al., 2017). The raw EEG data was first visually inspected, and portions of data outside of the putt periods and characterized by noise spread across all electrodes (due to transient changes in electrode impedance related to participants movements) were discarded. The electrodes (with the exception of prefrontal sensors FP1, FPz, and FP2) displaying abnormal power spectral activity (+/− 3 SD from mean signal recorded across included electrodes) were spherically interpolated using neighboring sensors signals. On average, 3.6 (SD = 1) electrodes were interpolated across participants. A 1 Hz to 30 Hz bandpass filter was applied (filter order: 1600, −6 dB, cut-off frequencies: 0.5 and 30.5 Hz) to the EEG signals. The data was re-referenced to the averaged electrodes. The filtered data then underwent a two step cleaning process aimed at parsing signals of artifactual sources (non-brain) from actual neural activity. In a first step, the filtered data was segmented into consecutive, non-overlapping one second segments. The signals of segments that were above or below three standard deviations from the overall mean of all segments were discarded. An extended infomax Independent Component Analysis (ICA; Makeig et al., 1996) was performed on the remaining data, with parameters adjustments to consider the rank deficiency of the data following average re-referencing and channel interpolation. The resulting Independent Components (ICs) were classified into categories using the IClabel (Pion-Tonachini et al., 2019). As a second step, the weights of the ICA decomposition were back projected to the filtered data (prior to rejecting one second segments). The ICs flagged as originating from muscles, eyes, line noise, and other non-brain sources by IClabel with a probability threshold above 70% were discarded. This resulted in the rejection of an average of 12 ICs (SD = 3). The proportion of remaining IC components after parsing non brain sources is in line with the guidelines proposed by Klug and Gramann (2021). This approach allows a more thorough but restrictive preprocessing to be applied as a first step (to ensure the quality of the ICA decomposition) and a less constraining approach to be employed during subsequent data processing steps. Following these processing steps, 3.5 s epochs were extracted (3 s pre contact and 500 ms post contact).

### 2.4 Data analysis

In all analyses statistical significance threshold was set at alpha = 0.05. To establish if there was a performance difference in QE, a paired *t*-test was conducted comparing mean QE duration for successful and unsuccessful putts.

An extraction of event-related spectral perturbation (ERSP; Makeig, 1993) features was performed through a time–frequency decomposition of the epoched data through the convolution of complex Morlet wavelets. The number of wavelet cycles ranged from 3 to 30 following a 0.8-step increase to estimate frequencies ranging from 3 to 30 Hz in 54 linearly spaced frequency steps. The spectral power at each frequency was baseline-corrected using a decibel (dB) transform relative to a baseline period of 500 ms (−3 to −2.5 s) prior to the period of interest (−2.5 s to 0 ms) performed on a single-trial basis (Grandchamp and Delorme, 2011). For ERSP analysis, the *a priori f*requency bands were selected based on the wider cognitive neuroscience and sporting literature, as follows: Theta (4–7 Hz), Alpha (8–12 Hz), Alpha Low (8–10 Hz), Alpha High (11–13 Hz), SMR (12–15 Hz) and Beta (12–30 Hz). The changes in overall power over the investigated frequency bands were then extracted for 5 consecutive time bins of 500 ms between the baseline period and the putt onset. In accordance with Del Percio et al. (2009) who adopted a neural efficiency framework approach, a series of Repeated Measures (2 × 5) ANOVAs with factors of Performance (Successful/Unsuccessful), Time (−2,500 to –2000 ms, −2000 to –1500 ms, 1,500 to –1000 ms, −1,000 to –500 ms, −500 ms to 0 ms) were separately carried out at each electrode cluster (Frontal: F3, Fz, F4/Central: C3, Cz, C4/Parietal: P3, Pz, P4), for each frequency band [theta, alpha (including low/high), SMR and beta].

For the Readiness Potential analysis, the continuous data sets were epoched around the onset of experimental events (−4,000 ms to 1,000 ms around putt onset). Consistent with the ERSP analysis, the epoched data were then baseline corrected by subtracting the mean voltage recorded within the 500-ms baseline period (−3 to −2.5 s) from the signal for each electrode and each trial. Averaging across epochs resulted in the creation of ERP waveforms for each individual electrode. These waveforms were then average across frontal, central and parietal clusters of electrodes. For each cluster, the readiness potential amplitude was computed as the mean voltage (in microVolts) of the ERP waveforms recorded within two successive 500 ms-long *a priori* time windows ranging from −1,000 to 0 ms prior to putting onset. Finally, statistical analyses were carried out at each electrode cluster (frontal/central/parietal) examining the extracted readiness potential features using a Repeated Measures (2 × 2) ANOVA with factors of Performance (Successful/Unsuccessful), Time (−1,000 to –500 ms, −500 to 0 ms) was separately carried out at each electrode cluster (frontal/central/parietal). All statistical testing was implemented in JASP version 0.6.13 (JASP Team, 2023).

## 3 Results

### 3.1 Performance

Performance was 69.71% (SD = 6.71%) for the sample of 28 participants. Performance for the sample of 12 participants included in the EEG sample, was 69.61% (SD = 7.37).

### 3.2 QE duration and performance

**Figure 2:**
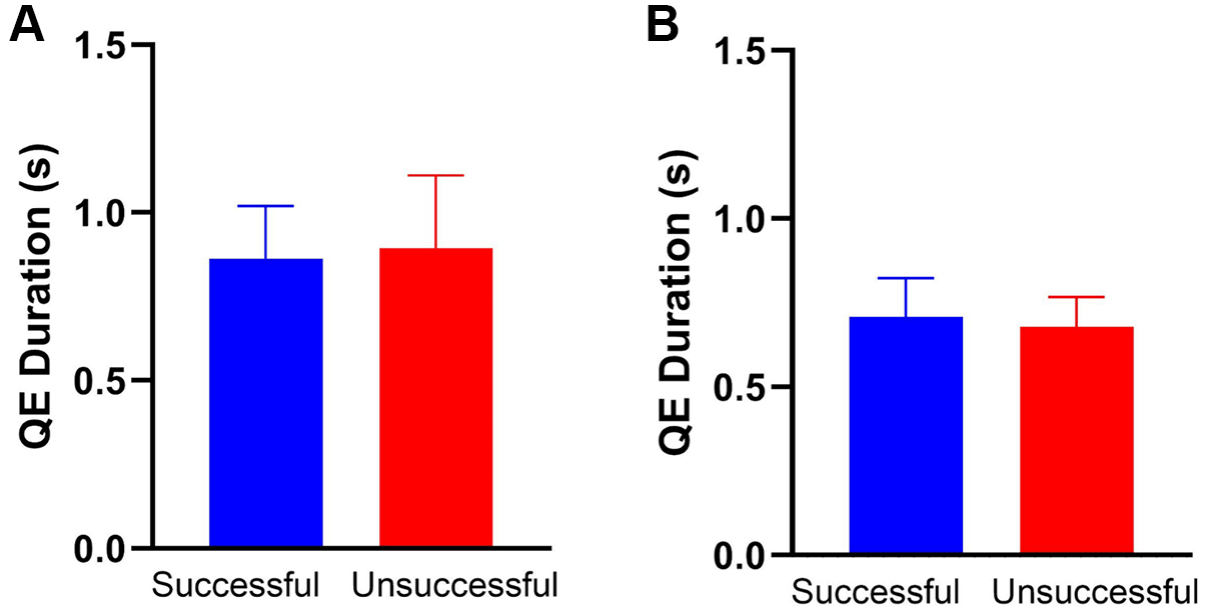
There are no performance-based differences in mean QE duration for either the full sample (*n* = 28, **A**) or the subset (*n* = 12, **B**) included in the EEG analysis. The error bars on both **(A,B)** are 95% CI.

The mean QE duration for successful putts was 0.86 s (*SD* = 0.357 s) and 0.89 s (*SD* = 0.486 s) for unsuccessful putts for the sample of 28 participants (Figure 2). There was no difference in mean QE duration [*t*_(21)_ *=* −0.670*, p* = 0.510, *d* = −0.143]. For the sample of 12 participants included in the EEG sample, average QE duration for successful putts (*M* = 0.71, *SD* = 0.18) and for unsuccessful putts (*M* = 0.68, *SD* = 0.14). There was no difference in mean QE duration [*t*_(21)_ *=* 0.454*, p* = 0.653, *d* = 0.140].

### 3.3 Neural activity and performance

**Figure 3:**
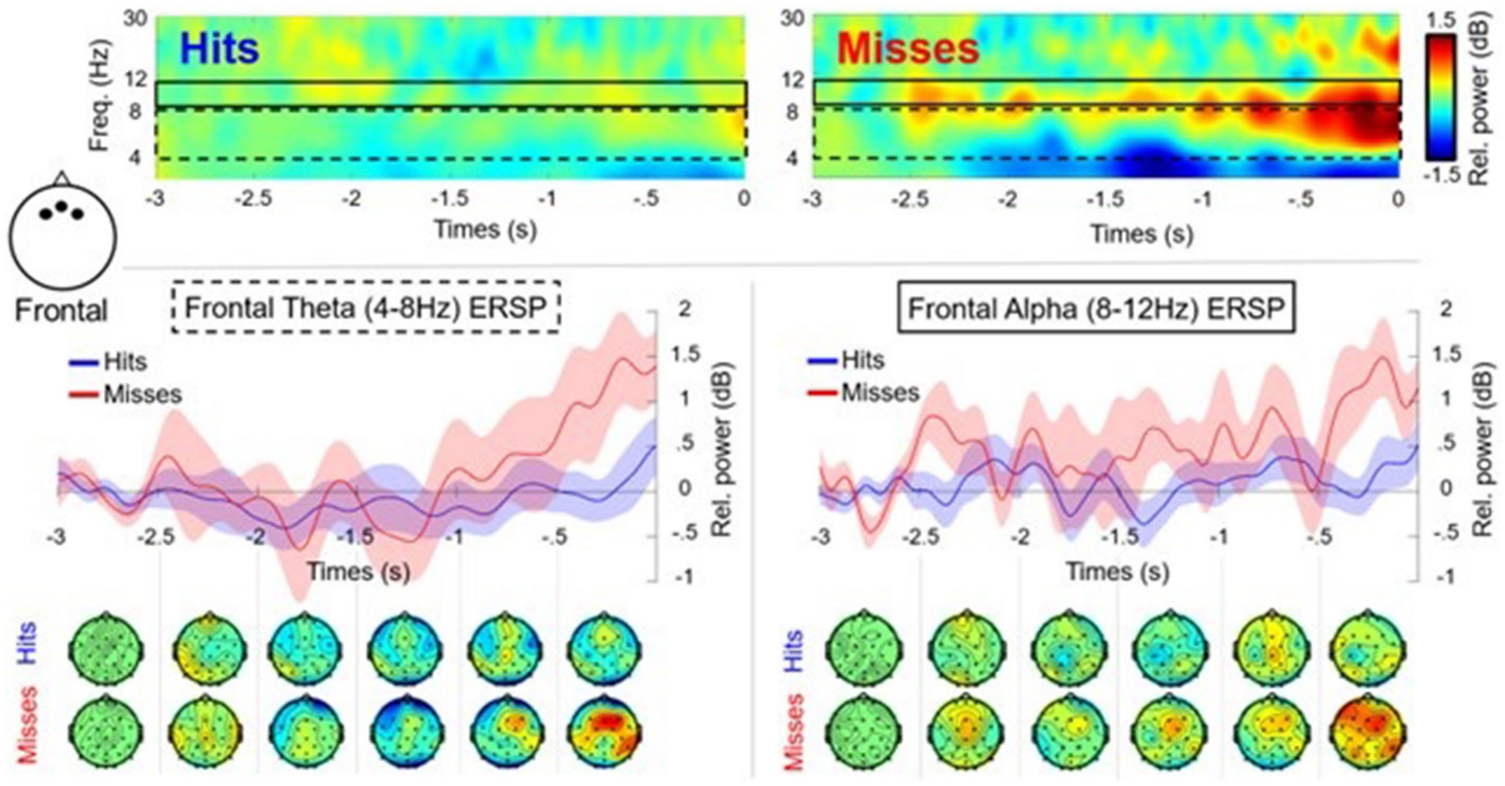
Time frequency plots and scalp maps showing theta (4–7 Hz) and alpha (8–12 Hz) oscillations for the frontal cluster (F3, Fz, F4) in the pre-motor preparation period (−3,000 ms to 0 ms) for successful (Hits: blue) and unsuccessful (Misses: red) putts (*n* = 12). There was a significant performance*time interaction for frontal theta power, but no other results were significant. The dashed black box highlights frontal theta activity with associated plot and topographic maps. The solid black line box shows frontal alpha activity with associated plot and topographic maps.

After the cleaning and processing stages, only 12 participants were retained with an average of 58 (SD = 8.91) successful trials and 25 (SD = 6.12) unsuccessful trials. The 500 ms post putt was removed from the analysis due to noise. The time frequency analysis, revealed a performance*time interaction [*F*(4, 44) = 3.125, *p* = 0.024, *n*^2^ = 0.041] for theta (Figure 3) in the frontal cluster (F3, Fz, F4). As seen in Figure 3, in the last three time windows (−1,500 ms to 0 ms) unsuccessful putts exhibited an increase of theta power in comparison to the theta power for the successful putts. None of the *post hoc* tests were significant within this RM-ANOVA, although the final time window (−500 ms to 0 ms/contact) was close (i.e., *p* = 0.07). The RM-ANOVA for theta at the central cluster (C3, Cz, C4) did not reveal a significant difference in performance or a performance*time interaction. The RM-ANOVA for theta at the parietal cluster (P3, Pz, P4) did not reveal a significant difference in performance or a performance* time interaction.

**Figure 4:**
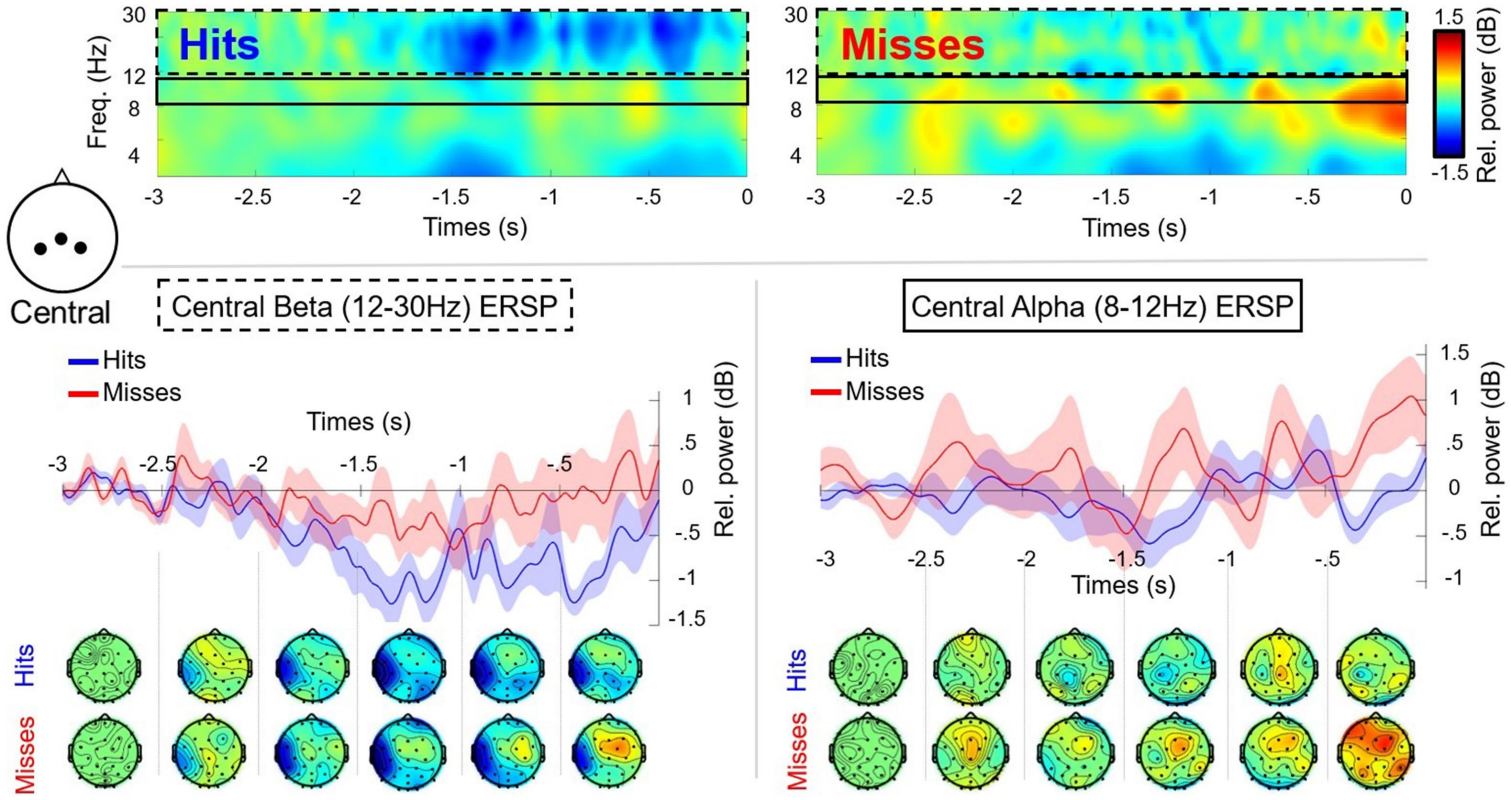
Time frequency plots and scalp maps showing alpha (8–12 Hz) and beta (12–30 Hz) oscillations for the central cluster (C3, Cz, C4) in the pre-motor preparation period (−3,000 ms to 0 ms) for successful (Hits: blue) and unsuccessful (Misses: red) putts (*n* = 12). There was a significant main effect for performance for central beta power, but no other results were significant. The dashed black box highlights central beta activity with associated plot and topographic maps. The solid black line highlights central alpha activity with associated plot and topographic scalp maps.

The RM-ANOVA comparing alpha (8–12 Hz) in frontal/central/parietal clusters, did not reveal any significant differences or interactions (Figures 3, 4). Additional analysis using low (8–10 Hz) and high (11–13 Hz) bands of alpha was also conducted for each of the frontal/central/parietal clusters. The analysis did not reveal any significant differences or performance*time interactions however, the main effect for performance for low alpha in the frontal cluster (F3, Fz, F4) was close (i.e., *p* = 0.06). The RM-ANOVA comparing the SMR in central/parietal clusters also revealed no significant differences or interactions. Regarding beta (central cluster), there was a main effect for performance [*F*(1,11) = 6.516, *p* = 0.027, *n*^2^ = 0.093], with a greater suppression (mean difference of −0.484 ± 0.190 dB) for successful putts in comparison to unsuccessful putts (Figure 4). There was no performance*time interaction. The RM-ANOVA revealed no significant differences in beta power at parietal sites (cluster P3, Pz, P4) or frontal (cluster F3, Fz, F4).

**Figure 5:**
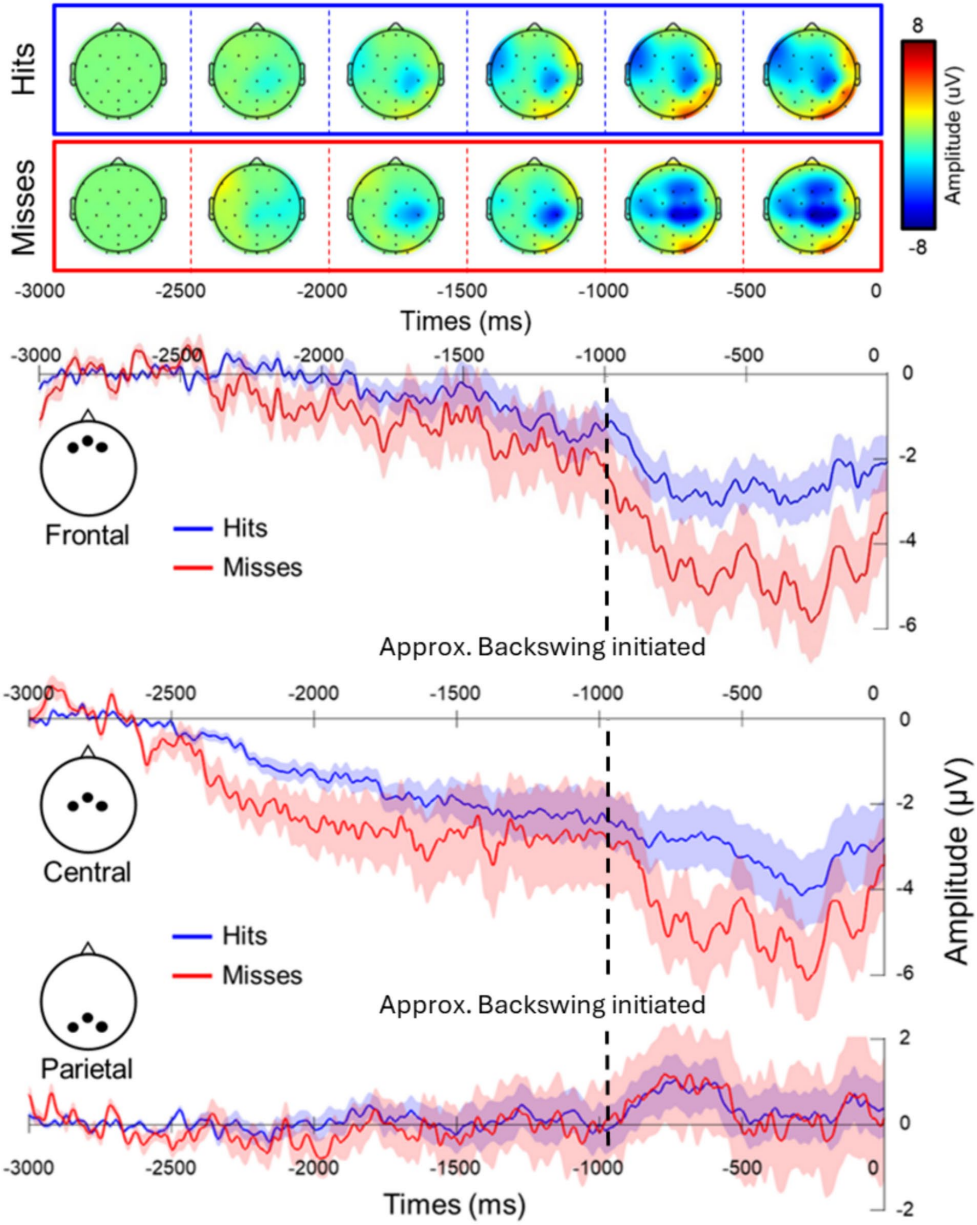
Differences in neural activity in the frontal cluster for successful (Hits: blue) and unsuccessful (Misses: red) putts for the readiness potential with associated plot and topographic scalp maps. The choice of trigger has limitations as the motor action (approximate initiation of the backswing represented by the black dashed line) can be seen as the trigger is aligned to contact (0 ms) not the initiation of the backswing.

Time analysis revealed a clear readiness potentials in both conditions at the frontal cluster with differences for successful shots in comparison to unsuccessful shots (mean difference = 1.706 ± 0.679 dB), as the RM-ANOVA revealed a main effect of performance [*F*(1, 11) = 6.304, *p* = 0.029, *n*^2^ = 0.248], with unsuccessful putts having a greater negative amplitude in comparison to successful ones (Figure 5). The RM-ANOVAs for central and parietal clusters did not reveal any significant effects or interactions.

## 4 Discussion

The current study aimed to address the hypotheses that successful performance can be distinguishable from unsuccessful performance based on QE duration and neural activity. We found there was no difference in mean QE duration based on performance (holed putts vs. missed putts). The mean durations of quiet eye (QE) phases alone may not reliably indicate expertise. Critically van Lier et al. (2010) and Mann et al. (2011) also did not find QE duration differed based on expertise. It is worth noting the QE durations were lower than the optimal QE duration 2–3 s recommended for putting (Vickers, 2007), highlighting the potential need for a training intervention to achieve optimal QE duration. Consistent with our findings, van Lier et al. (2010) found without training, golfers had QE duration less than the recommended duration. By integrating eye tracking with EEG data, a deeper understanding can be gained regarding why identical QE durations can result in either successful or unsuccessful putts, as well as shedding light on the timings of optimal QE and the merits of teaching a QE intervention by examining the 3 s prior to contact. For instance, our findings reveal that successful putts exhibit distinct neural activity profiles compared to unsuccessful putts. Successful putts revealed a greater suppression compared to unsuccessful putts. The greater suppression in successful putts may signify activation of sensorimotor networks, indicating enhanced movement planning.

Additionally, performance differences in theta frequency were noted in the frontal region, with successful putts displaying a tendency for lower theta power, particularly in the final time window, compared to unsuccessful putts. Increased theta power for unsuccessful putts may indicate hesitation or the need for an adjustment to the motor plan prior to execution, resulting in inefficiency and extra cognitive demands, in line with the neural efficiency framework (Del Percio et al., 2009). These findings are also consistent with the findings of a meta-analysis by Filho et al. (2021) examining self-paced sports that provided support for the neural efficiency framework, specifically a decrease in theta was linked to optimal performance. From an applied perspective, our findings shed light on the timings and nuances to the process of putting outlined by Mann et al. (2011), when putting, players must maintain the intended putt line in working memory while focusing on the ball. They must then activate a motor program to accurately strike the ball with the necessary force and direction for the desired outcome (Mann et al., 2011). If there is a disruption in motor planning or lack of commitment to the first intended motor plan, then this will disrupt the performance. Here, we found greater suppression of beta activity in the central region during successful performance. In support of our findings, Tzagarakis et al. (2010) found the power decrease for beta during motor preparation was scaled relative to uncertainty, with the greatest reduction of power associated with certainty. Combined with the aforementioned theta findings above, this offers further support that unsuccessful putts are associated with uncertainty and hesitation, as the suppression (reduction in power) for unsuccessful putts was less than successful putts. The greater suppression for successful putts is considered an index of cortical activation (Tzagarakis et al., 2010; Kilavik et al., 2013). While it may not indicate more efficient activation on a neural level, we propose that greater beta suppression leads to enhanced preparation, which could be considered a form of increased efficiency. Additionally, we speculate that beta suppression may offer insight into the mechanisms underpinning QE duration, especially as the beta suppression onset timings for successful putts are consistent with recommended QE duration of 2–3 s (Vine et al., 2011). Furthermore, the monitoring aspects of theta may also offer insight towards the mechanism underpinning the proposed role of QE duration in continuous monitoring and online control (Gonzalez et al., 2017). Taking the findings together, we propose that neural activity may provide plausible insights into the mechanisms behind QE and how and why identical QE durations may lead to both successful and unsuccessful putts. Our findings offer working hypotheses and tentative explanations towards clarifying ambiguities regarding the efficacy of QE recommendations. Moving forwards, further research is required to support these claims.

Unexpectedly, our study did not find any performance-based difference in alpha power. These findings contrast with other studies where performance-based differences in alpha power were reported (Babiloni et al., 2008; Baumeister et al., 2008; Cooke et al., 2014) and do not align with the neural efficiency framework. Our research contributes to the ongoing discourse on the inconsistencies observed in alpha studies related to golf putting (Park et al., 2015). We advocate for further investigation into alpha power and performance during the pre-preparatory phase. Consistent with Pfurtscheller et al. (2003) we found the low and high alpha bands, (in our case low alpha) were more sensitive to performance differences so we recommend future research continues to adopt this approach. To facilitate comparison across studies, we recommend adopting standardized methodologies, including consistent epochs, task design, and data analysis approaches.

Our findings revealed that it is possible to observe performance-based differences, reflected in the amplitude of the readiness potential, with successful putts having a less negative amplitude in comparison to unsuccessful putts, in line with the neural efficiency framework (Del Percio et al., 2009). This finding offers support for the proposal that successful putts are associated with reduced response programming demands (Wright et al., 2012) requiring less energy (Di Russo et al., 2005). These findings may seem contradictory to the beta findings presented above, but recent research has suggested that beta and RP could reflect different phenomena within the movement preparation processes (Gavenas et al., 2023; Parés-Pujolràs et al., 2023). We recommend that future research explores how beta desynchronization in the motor cortex relates to the RP.

### 4.1 Limitations and future research

We propose the EEG findings are not trivial, as both the definition of experimental event in such a naturalistic context and the processing of neural data acquired while whole body motion was unrestricted posed substantial challenges. To address these challenges and to maintain good signal to noise data, we used a rigorous process for cleaning the EEG data and this did result in a high loss of data. Our study is not without limitations, especially as it is a single study with 12 participants and we would recommend further research with more participants, especially when using repeated measures ANOVAs. We used an acoustic trigger to timestamp the moment the club made contact with the ball, so the movement had to occur during the epoch, meaning that we could not accurately detect the initiation of the backswing. We suggest modifying the EEG data time stamping method to precisely capture both the contact point and initiation of movement, crucial for investigating readiness potentials, potentially utilizing lightweight accelerometers on equipment like clubs, if feasible without affecting stroke kinematics. Additionally, we recommend future research utilizes recent technology advancements that allow for the collection of synchronized eye tracking and EEG data acquisition and time stamp the EEG data through fixations (Ladouce et al., 2022). This approach would allow for the working hypotheses of the mechanisms underpinning QE to be explored in detail as a direct analysis of QE duration and EEG can be undertaken. For more detail on this approach and the potential of synchronized eye tracking and EEG data and the feasibility, including outlining current challenges with this approach, are discussed in Ladouce et al. (2017). We would also encourage future researchers to consider participant recruitment, design and trial numbers for a good signal-to-noise ratio. Recruiting a highly skilled sample has clarified some of the ambiguities in prior research regarding the directionality of power and we believe future research with an increased sample size would continue to strengthen the research in this area. Despite the challenges, we believe this study paves the way for further investigation of the neural correlates of sporting performance by showcasing methods to effectively capture neural dynamics of action planning in applied sporting contexts.

### 4.2 Practical implications

This study unveils the challenges encountered during EEG data collection in a practical scenario and proposes solutions to overcome these hurdles. While highlighting the benefits of this approach, it stresses the importance of methodological rigor, especially in EEG data analysis. Golf putting may serve as an applied context to delve deeper into the relationship between beta and MRCPs, specifically readiness potentials, to offer fruitful theoretical insights. Furthermore, the findings demonstrate tentative evidence to guide the efforts to unveil the mechanisms behind QE and clarify its effectiveness (Williams, 2016). We acknowledge that our findings stem from a single study, underscoring the need for future longitudinal studies with consistent methodological approaches to establish a more robust understanding of the relationship between neural activity and expertise. We understand the complexity involved in such research endeavors, both in terms of time investment and methodological intricacies. Nevertheless, we encourage researchers to embrace the multifaceted nature of the sporting domain (Bishop, 2008) when investigating markers of cognitive-motor expertise in golf putting and strive to develop practical recommendations. Only then do we believe it would be appropriate to provide recommendations for athletes, coaches, and practitioners.

## Data availability statement

The original contributions presented in the study are publicly available. This data can be found here: DOI 10.17605/OSF.IO/D6483.

## Ethics statement

The studies involving humans were approved by University of Stirling (School of Psychology and Natural Sciences). The studies were conducted in accordance with the local legislation and institutional requirements. The participants provided their written informed consent to participate in this study. Written informed consent was obtained from the individual(s) for the publication of any potentially identifiable images or data included in this article.

## Author contributions

LC: Writing – original draft, Writing – review & editing, Conceptualization, Data curation, Formal analysis, Funding acquisition, Investigation, Methodology, Project administration, Visualization. GA: Methodology, Writing – review & editing. SL: Conceptualization, Formal analysis, Visualization, Writing – review & editing. DK: Conceptualization, Methodology, Writing – review & editing. MB: Formal analysis, Methodology, Writing – review & editing. AH: Supervision, Writing – review & editing. DD: Conceptualization, Funding acquisition, Supervision, Writing – review & editing.
